# Mastitis in Autoimmune Diseases: Review of the Literature, Diagnostic Pathway, and Pathophysiological Key Players

**DOI:** 10.3390/jcm9040958

**Published:** 2020-03-30

**Authors:** Radjiv Goulabchand, Assia Hafidi, Philippe Van de Perre, Ingrid Millet, Alexandre Thibault Jacques Maria, Jacques Morel, Alain Le Quellec, Hélène Perrochia, Philippe Guilpain

**Affiliations:** 1 St Eloi Hospital, Department of Internal Medicine and Multi-Organic Diseases, Local Referral Center for Systemic and Autoimmune Diseases, 80 Avenue Augustin Fliche, F-34295 Montpellier, France; radjiv.goulabchand@chu-nimes.fr (R.G.); a-maria@chu-montpellier.fr (A.T.J.M.); a-lequellec@outlook.fr (A.L.Q.); 2 Internal Medicine Department, Caremeau University Hospital, 30029 Nimes, France; 3 Montpellier School of Medicine, University of Montpellier, 34967 Montpellier, Francei-millet@chu-montpellier.fr (I.M.); j-morel@chu-montpellier.fr (J.M.); h-perrochia@chu-montpellier.fr (H.P.); 4 Inserm U1183, Institute for Regenerative Medicine and Biotherapy, St Eloi Hospital, 80 Avenue Augustin Fliche, 34295 Montpellier, France; 5 Gui de Chauliac Hospital, Pathology Department, 80 Avenue Augustin Fliche, 34295 Montpellier, France; 6 Pathogenesis and Control of Chronic Infections, Univ Montpellier, INSERM, EFS, Montpellier University Hospital, 34394 Montpellier, France; p-van_de_perre@chu-montpellier.fr; 7 Lapeyronie Hospital, Montpellier University, Medical Imaging Department, 371 Avenue du Doyen Gaston Giraud, 34295 Montpellier, France; 8 Department of Rheumatology, CHU and University of Montpellier, 34295 Montpellier, France

**Keywords:** mastitis, breast lymphocytic infiltrates, mammary duct ectasia, granulomatous mastitis, vasculitis, IgG4-related disease

## Abstract

Mastitis frequently affects women of childbearing age. Of all the pathological breast conditions requiring specific management, autoimmune mastitis is in the third position after infection and breast cancer. The aim of this literature review was to make a comprehensive description of autoimmune diseases targeting the mammary gland. Four main histological patterns of autoimmune mastitis are described: (i) lymphocytic infiltrates; (ii) ductal ectasia; (iii) granulomatous mastitis; and (iv) vasculitis. Our literature search found that all types of autoimmune disease may target the mammary gland: organ-specific diseases (diabetes, thyroiditis); connective tissue diseases (such as systemic erythematosus lupus or Sjögren’s syndrome); vasculitides (granulomatosis with polyangiitis, eosinophilic granulomatosis with polyangiitis, giant cell arteritis, polyarteritis nodosa, Behçet’s disease); granulomatous diseases (sarcoidosis, Crohn’s disease); and IgG4-related disease. Cases of breast-specific autoimmune diseases have also been reported, including idiopathic granulomatous mastitis. These breast-limited inflammatory diseases are sometimes the first symptom of a systemic autoimmune disease. Although autoimmune mastitis is rare, it is probably underdiagnosed or misdiagnosed. Early diagnosis may allow us to detect systemic diseases at an earlier stage, which could help to initiate a prompt, appropriate therapeutic strategy. In case of suspected autoimmune mastitis, we hereby propose a diagnostic pathway and discuss the potential pathophysiological pathways leading to autoimmune breast damage.

## 1. Introduction

Mastitis is a common pathological condition among women of childbearing age. It is an important diagnostic challenge as breast cancer and infections (notably tuberculosis) must be sought and rejected. Autoimmune mastitis belongs to a third group of etiologies. This group has recently been described in the medical literature and includes a wide range of autoimmune diseases [1,2,3]. Recent case reports are numerous, suggesting that this condition is underdiagnosed or misdiagnosed [1,2,3,4,5]. The clinical spectrum of autoimmune mastitis is broad: while some patients are asymptomatic, others have severe, recurrent breast inflammation, painful nodules, nipple discharge or retraction, and/or lymphadenopathy.

In this article, we distinguish four main histological patterns of autoimmune mastitis: (i) lymphocytic infiltrates; (ii) ductal ectasia; (iii) granulomatous mastitis; and (iv) vasculitis. Although these four histopathological patterns are not specifically related to one particular autoimmune disease, they provide an indication for the physician’s diagnostic pathway, especially when extramammary symptoms are rare or not yet detectable. It is important to note that certain autoimmune diseases share several of these four patterns, a point which will also be developed further.

Because autoimmune mastitis and cancer both involve women of varying ages and as suspected cancer is an anxiety-provoking situation for patients, physicians must establish a precise diagnostic strategy to avoid repeated, tissue-damaging breast biopsies. Consequently, the aim of our work was to describe the different types of autoimmune mastitis, depicting their clinical, biological, and histological features, causes, and therapeutic options.

## 2. Methods

We conducted a literature search in Pubmed in order to review all kinds of histological breast findings associated with autoimmune diseases. We used the following keywords: “mastopathy”; “breast diseases”; “lymphocytic mastitis”; “granulomatous mastitis”; “diabetic mastopathy”; “sclerosing lymphocytic lobulitis”; “thyroiditis”; “autoimmune disease”; “Sjögren’s syndrome”; “systemic erythematosus lupus”; “granulomatosis with polyangiitis”; “eosinophilic granulomatosis with polyangiitis”; “idiopathic granulomatous mastitis”; “sarcoidosis”; “IgG4-related disease”; “aseptic cutaneous abscesses”; “autoinflammatory disease”. We selected all articles written in English that described autoimmune mastitis.

For didactic purpose and because the first-line investigation for breast disease comprises a breast biopsy and pathological analysis, we decided to develop our description of autoimmune mastitis according to the four above-mentioned histological patterns. These aspects can guide physicians to the right diagnosis, even if none of them are specific to one disease (and one disease can have several histological patterns in the breast).

## 3. Breast Lymphocytic Infiltrates

**Figure 1:**
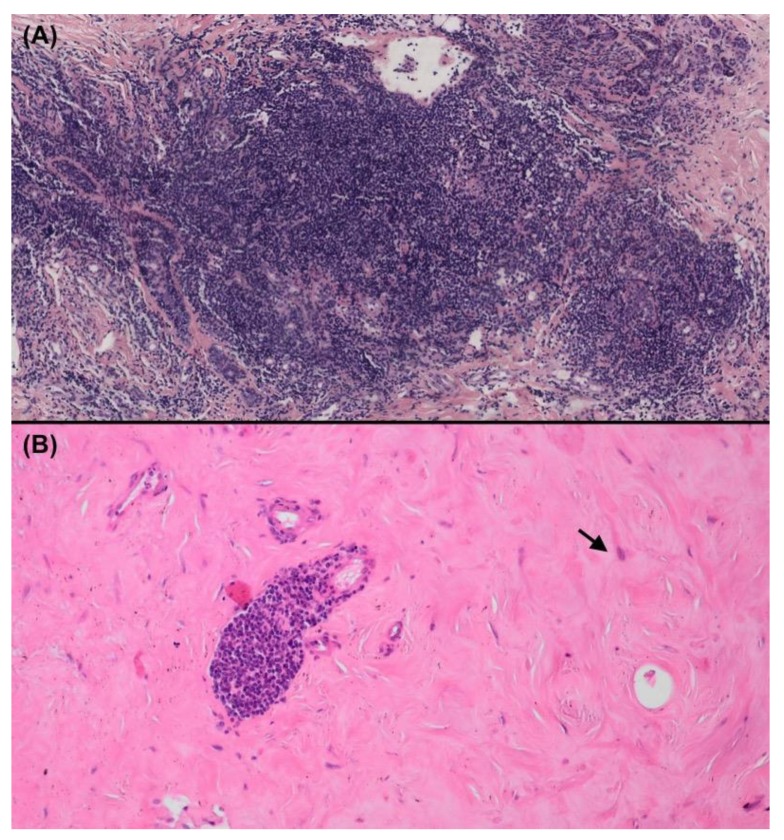
Lymphocytic infiltrates of the breast. (**A**) Dense, pseudonodular, lymphocytic infiltrates associated with a diagnosis of Sjögren’s syndrome mastitis; (**B**) Dense keloid-like fibrosis with perivascular lymphocyte infiltrates and prominent myofibroblasts in keloid stroma (arrow) associated with a diagnosis of diabetic mastopathy (Hematoxylin and eosin staining, magnification × 100).

Lymphocytic infiltrates are common within breast tissue (Figure 1). A normal breast gland contains myeloid and lymphoid cells, mainly located within the lobules rather than in breast fat or breast stroma. A higher proportion of cytotoxic T cells and dendritic cells than T CD4+ and B CD20+ cells is observed in a normal breast and they are mainly located within the epithelium [6].

Consequently, some authors have proposed a cut-off of 50 or 100 lymphocytes per lobule as the criterion for defining abnormal lymphocytic breast infiltrates [7,8]. Abnormal lymphocytic infiltrates in the breast are also characterized by a decrease in T cytotoxic lymphocyte and dendritic cell populations, which are also less present within the glandular epithelium [6]. Epidemiological data depicting the etiologies of lymphocytic infiltrates of the breast are rare because they are multiple. Although diabetes is the most frequently depicted etiology, other autoimmune diseases should be investigated, such as thyroiditis, Sjögren’s syndrome, lupus, IgG4-related disease, or Biermer’s disease. It should be noted that in our literature review, we excluded lymphocytic infiltrates related to breast lymphomas, including anaplastic large-cell lymphomas diagnosed in the context of breast implants [9].

### 3.1. Diabetes

Diabetic lymphocytic mastitis, also known as “diabetic mastopathy” or “sclerosing lymphocytic lobulitis”, mainly arises among Type 1 diabetic patients or Type 2 diabetic patients requiring insulin treatment. It can also occur in some patients who do not receive insulin treatment [10]. The true frequency of diabetic mastitis might be higher than expected, as suggested by a retrospective study with 70% (17/24) of Type 1 diabetic patients exhibiting sclerosing lymphocytic lobulitis [11]. Mastitis may also occur in male patients with gynecomastia [12,13].

Diabetic mastopathy usually affects patients around the age of 40 and after a long disease duration [14,15]. Thus, a long-term history of Type 1 diabetes represents a strong criterion for clinical diagnosis. Physical examination can show painless but hard, irregular, easily movable breast masses, solitary or multiple, and bilateral in some cases. Some specific clinical and radiological findings (ultrasonography, MRI) have been established in order to allow physicians to suggest a diagnosis of diabetic mastopathy [16,17,18,19]. However, since diabetic mastopathy can mimic breast neoplasms, a histological diagnosis is often required [20,21,22,23,24,25].

Histologically, diabetic mastopathy shows lobulitis and vasculitis, keloidal fibrosis, lobular atrophy, and varying degrees of epithelioid fibroblasts. Lymphocytic infiltrates are typically circumscribed aggregates of small lymphocytes surrounding vessels, lobules, and periductal tissues. In order to distinguish diabetic mastopathy from other autoimmune causes, some authors suggested that dense keloid-like fibrosis containing peculiar epithelioid cells are pathognomonic of diabetic mastopathy [26] (Figure 1B). Some authors have even suggested that a tight circumscription of the lymphoid infiltrate around lobules is specific to diabetic mastopathy, whereas a loose distribution of lymphocytes among mammary stroma is suggestive of other causes [11]. Immunohistochemical features of inner white cells have also been reported: B-cell lymphoid infiltrates are predominant, with no individualized monoclonal populations [15,26,27,28,29].

Several pathophysiological hypotheses have been evoked concerning diabetic mastopathy, including autoantibodies targeting mammary cell antigens. Miura and coll. described one case of lymphocytic mastopathy in a patient with a 31-year history of Type 2 diabetes [30] and demonstrated the presence of circulating anti-insulin autoantibodies with cross-reactivity towards breast duct epithelium. Such autoantibodies might be linked to the onset of inflammation within the breast tissue and trigger clinical mastitis flares. Another hypothesis involves sustained hyperglycemia resulting in an accumulation of advanced glycosylated end products, which are highly immunogenic and may favor cytokine production, B-cell lymphocyte proliferation, and matrix remodeling, all leading to breast inflammation.

### 3.2. Thyroiditis

Thyroid diseases represent the second group of autoimmune diseases associated with lymphocytic mastitis. In their literature review, Boullu and coll. reported 11 cases with a histological pattern of “diabetic mastopathy” and associated autoimmune thyroiditis [14,27]. One case of sclerosing lymphocytic lobulitis occurring at the time of diagnosing Graves-Basedow disease and another case during Hashimoto’s thyroiditis were also reported [31,32].

### 3.3. Sjögren’s Syndrome

Sjögren’s syndrome (SjS) can target the mammary gland. Before we recently described this specific involvement, only two cases had been reported [3]. First, a 49-year-old female patient had xerostomia and bilateral mastitis within 8 months of beginning tiopronine (Acadione^®^) treatment for rheumatoid arthritis [33]. Anti-Ro/SSa antibodies positivity and the salivary gland biopsy findings, revealing lymphocytic aggregates with interstitial fibrosis and acinar atrophy, were consistent with a diagnosis of SjS. The breast biopsy revealed significant lymphocytic infiltrates. The authors suggested the potential role of the recently introduced tiopronine treatment on the onset of mastitis and also discussed the possible implication of SjS on the pathophysiology of mastitis. The second case concerned the observation of lymphocytic mastitis preceding the diagnosis of SjS in a 43-year-old woman. The patient had a 2 cm × 5 cm lump in her right breast, characterized histologically by dense perilobular polyclonal lymphocytic infiltrates [34]. Two months later, arthralgia, xerostomia, eye itching, chronical bronchitis, positive ANA (1:640), and positive anti-Ro/SSA antibodies (815 U/mL) led to the diagnosis of SjS [34]. These two cases suggested a relationship between inflammatory mastopathy and SjS and are in line with our recently published case series [3]. From a study-population of nine women, we described the clinical and histological spectra of SjS-associated mastitis. While these patients had few systemic symptoms related to SjS, they presented breast symptoms (recurrent mastitis, nipple discharge, or breast lumps) concomitantly or before the onset of the systemic symptoms of Sjögren’s disease. Interestingly, the histological patterns of breast and salivary glands were close: histological patterns of SjS mastitis showed lymphocytic infiltrates, which were sometimes very compact and very close to the classical “focus” found in the salivary gland (Figure 1A). We also found duct ectasia in both mammary and salivary glands (as developed in §4).

The anatomical, histological, and physiological roles of salivary glands and mammary glands are similar as they both belong to the effector sites of the associated mucosal immune system. Their ductal cells share common receptors such as the SSa antigen [35]. This explains why autoimmune diseases such as SjS might target these two glands concomitantly and this is perfectly in line with the literature which considers Sjögren’s syndrome as “autoimmune epithelitis” [36,37].

To our knowledge, eleven cases of Sjögren-associated mastitis (lymphocytic infiltrates and/or duct ectasia) have been described so far. Sjögren’s syndrome mastitis is in line with the classical involvement of exocrine glands in this disease. It should also be noted that four cases of Sjögren-associated granulomatous mastitis have been reported (§5.3).

### 3.4. Lupus

Lupus mastitis is a rare benign inflammation of the deep subcutaneous adipose tissues of the breast. It is part of lupus panniculitis, but is called lupus mastitis when involving the breast glands. So far, 27 cases have been reported in the literature: Kinonen and coll. reviewed 22 cases and 6 additional cases have since been reported [38,39,40,41,42,43,44].

Lupus mastitis usually affects women around the age of 40 with a pre-established diagnosis of lupus. Clinically, epidermal manifestations may be observed (atrophy, erythema, lipoatrophy, hypertrichosis, or ulceration). The clinical presentation is heterogeneous, ranging from firm to hard palpable masses as observed in patients with diabetic mastopathy. The radiographic features of lupus mastitis may mimic breast cancer (breast density associated with calcifications). Therefore, a precise definition of the clinical and radiological features of lupus mastitis have been depicted in order to define the diagnosis, avoiding aggressive surgery or tissue-damaging biopsies which might potentially trigger a relapse of lupus [1]. The typical histological hallmark of lupus mastitis is hyaline fat necrosis. Dense lymphoid infiltrates, fibrosis, and even calcifications may also be observed. Linear deposition of IgG and C3 along vessel basement membranes and the dermo-epidermal junction can be revealed by immunofluorescence. Lymphocytic infiltrates in lupus are distinguishable from those observed in diabetic mastopathy as they are more profuse and present a lobular distribution [39].

In addition to the well-characterized cases of lupus, antinuclear antibodies (ANA) were detected in 50/78 patients with non-lactational mastitis in the study by Xu and coll. [45]. However, since these patients did not fulfill the diagnostic criteria for systemic lupus and histological involvement was not described in this work, the link with lupus remains uncertain.

### 3.5. Mixed Connective Tissue Disease

Breast involvement has also been reported in one patient with mixed connective tissue disease [46]. In this case, a breast screening strategy led to the discovery of suspicious groups of coarse, sheet-like calcifications. The breast biopsy revealed panniculitis (fat necrosis adjacent to breast tissue) and inflammation within breast tissue (lymphocytic infiltrates and vasculitis).

### 3.6. IgG4-Related Breast Disease

Recently, unspecific tissue inflammation (such as pancreatitis, retroperitoneal fibrosis, and primary suppurative cholangitis) was reported in association with misdiagnosed IgG4+ disease, known as “IgG4-related sclerosing autoimmune disease”. Histologically, IgG4-related disease is characterized by the following findings: dense lymphoplasmacytic infiltrates; fibrosis arranged at least focally in a storiform pattern; and obliterative phlebitis. An increased number of IgG4+ plasma cells within the tissue is also part of the classical histological pattern, but not essential to diagnosis.

Forty-seven cases of lymphocytic infiltrates in the mammary glands related to IgG4-related disease have been reported so far [2,4,47,48,49,50,51,52,53,54,55,56]. The majority of patients were women presenting with unilateral or bilateral painful mammary pseudotumors, mimicking breast cancer. In addition to classical histological patterns of IgG4 disease, loss of breast lobules is also described [48]. As observed in other targeted organs, the IgG4/IgG-secreting plasma cell ratio is higher (a small proportion of IgG4-secreting plasma cells is not specific enough to allow the diagnosis of IgG4-related disease). Hence, Cheunk and coll. reported 4 cases of IgG4-related mastitis (lymphoplasmocytic infiltrates with stromal sclerosis and loss of breast lobules) in which the lymphoid population consisted of both B and T cells, and the IgG4+/IgG plasma cell ratio was between 49% and 85% [48]. We may also note that IgG4-related mastitis has been reported in certain patients with only one other organ involved (autoimmune pancreatitis or orbital mass) [52,57]. Moreover, the breast gland was the only organ targeted by IgG4-related disease, in the absence of other classical involvements (pancreas, salivary glands, or periorbital tissue) in some cases [58].

### 3.7. Biermer’s Disease

In their review of the literature, Boullu and coll. reported 2 cases of anti-parietal cell antibodies detected in 49 cases of lymphocytic mastitis. However, the evaluation criteria for the diagnosis of acquired pernicious anemia (Biermer’s disease) were not reported [14].

### 3.8. Primary Lymphocytic Infiltrates Involving the Breast

Some cases of lymphocytic infiltrates involving the breast have been reported in the absence of defined autoimmune diseases. For example, our literature review identified 24 cases of “primary” lymphocytic mastitis out of 73 reported cases [12,15,50,59]. Primary lymphocytic mastitis is a diagnosis of exclusion and physicians must rule out other pathological conditions, particularly cancer and lymphomas. However, our literature review and our own experience (particularly in SjS) suggest that extra-mammary systemic symptoms or biological markers associated with autoimmune diseases can occur a long time after breast symptoms occurrence. Certain cases of so-called “primary” lymphocytic infiltrates of the breast should, therefore, be considered as the autoimmune disease’s first lesion, after discarding breast cancer or infection. Hence, follow-up of these patients exhibiting “primary” lymphocytic infiltrates involving the breast could be prolonged and include repeated clinical, immunological, or radiological investigations depending on the evolution of symptoms [28,60].

## 4. Mammary Duct Ectasia

**Figure 2:**
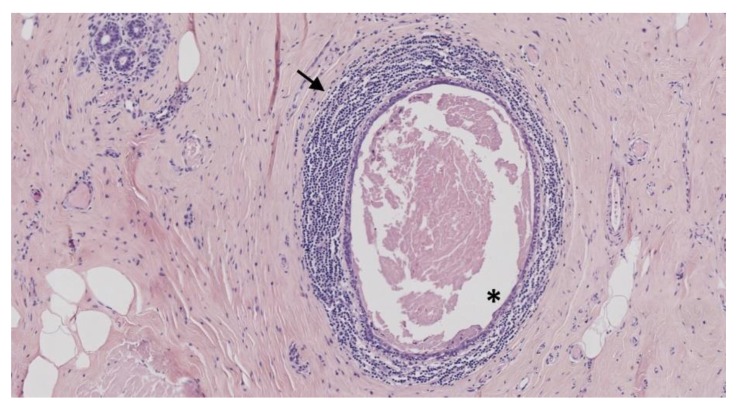
Mammary duct ectasia. Ductal ectasia (asterisks). Periductal lymphocytic infiltrates (arrow) (Hematoxylin and eosin staining, magnification × 100).

Mammary duct ectasia (MDE) is a benign condition characterized by dilatation of the retro-areolar mammary ducts (Figure 2). Its frequency is very variable depending on the diagnostic procedure used (clinical, radiological, or histopathological) [61]. MDE usually occurs in women younger than 30 years or around menopause, but some cases in children and male patients have been reported [62,63]. MDE is commonly considered as an inflammatory condition of the breast [64]. Dixon and coll. described a large histopathological series of mammary duct ectasia, suggesting that periductal mastitis preceded duct ectasia: indeed, younger patients had more periductal inflammation around non-dilated ducts, whereas older patients mainly had ductal dilatation [65]. A hypothetical mechanism is that periductal inflammatory infiltrates, consisting of lymphocytes and histiocytes, may lead to subsequent destruction of the duct’s basal membrane, leading to ductal dilatation. This pathophysiological process might be triggered by recurrent breast infections [66]. However, ductal dilatation has also been described as a normal involution of breast tissue in women over 80 years old [67,68].

Indeed, the pathophysiology of MDE is unclear and several potential causes have been suggested. Environmental factors (such as smoking, with conflicting data), local factors (inflammation linked to breast infections), or hormonal status (lactation and pregnancy, or hyperprolactinemia) may all play a role in the origin of MDE [66,68]. Finally, ductal dilatation may also be secondary to an obstruction caused by papilloma or carcinoma. Thus, cancer diagnosis must be ruled out before considering an autoimmune reason for MDE.

The present systematic review of the literature identified only a few cases of defined autoimmune or inflammatory diseases associated with mammary duct ectasia. One case of histologically proven MDE was reported in association with Behçet’s disease [69] and two other cases with SjS [3]. One case of duct dilatation diagnosed as MDE was also found among patients with thyroid disease [70]. These associations are rare and their pathophysiological links remain hard to establish. In addition, since MDE is sometimes considered as a normal involution of the breast gland, its potential relationship with concomitant autoimmune processes is often ignored.

## 5. Granulomatous Mastitis

**Figure 3:**
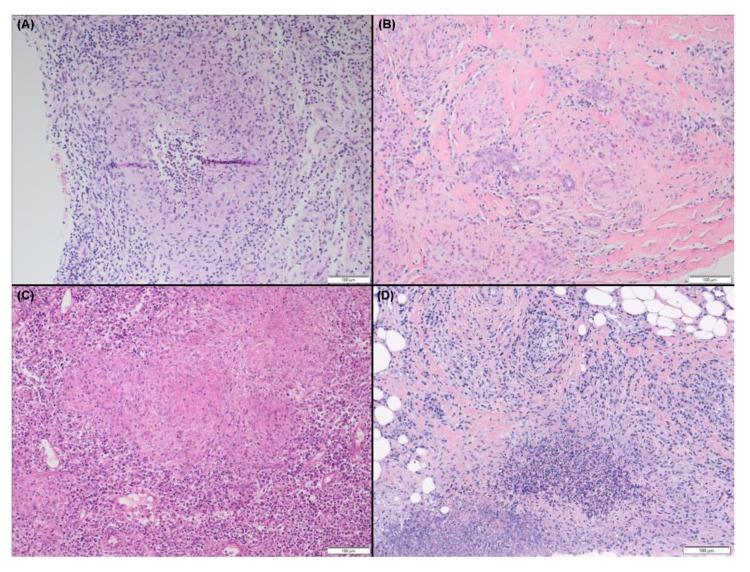
Different histological patterns of granulomatous mastitis. (**A**) Granulomatous mastitis leading to a diagnosis of idiopathic granulomatous mastitis with polymorphonuclear neutrophil micro abscess; (**B**) Non-necrotizing epithelioid and gigantocellular granulomas involving a lobule with lymphoplasmacytic reaction and fibrosis, leading to a diagnosis of breast sarcoidosis; (**C**) Granulomatous mastitis with caseous necrosis leading to a diagnosis of tuberculous mastitis; (**D**) Granulomatous mastitis with neutrophils micro abscess in a 34-year-old patient with a granulomatous with polyangiitis vasculitis with lung and skin involvement (Hematoxylin and eosin staining, magnification × 100).

Granulomatous mastitis is defined histologically by lobulocentric non-necrotizing granulomas, associated with epithelioid histiocytes (Figure 3A). Two main groups of breast diseases may share granulomatous patterns: breast cancer and infectious diseases. Among granulomatous breast infections, tuberculosis mastitis is the most common [71] (Figure 3C), followed by various infections (cat-scratch disease, Proprionibacterium, or Corynebacterium) [72,73]. A third etiologic group is represented by autoimmune diseases and includes well-defined clinicopathological granulomatous diseases such as Crohn’s disease, granulomatosis with polyangiitis (Wegener’s disease), or sarcoidosis, as well as more recently described conditions such as IgG4-related disease, systemic erythematosus lupus, or SjS [74,75,76]. Once all these causes have been excluded, Idiopathic Granulomatous Mastitis (IdGM) can be considered [71,77,78]. As discussed below, this benign and well-described breast disease may also be considered as an autoimmune disease limited to the breast.

### 5.1. Granulomatous Mastitis Associated with Systemic Granulomatous Diseases

#### 5.1.1. Inflammatory Bowel Diseases

Crohn’s disease is a rare but classical cause of mastitis and may present as a breast mass mimicking an abscess, histologically characterized by granulomatous inflammation with eosinophilic infiltrates [79]. This is consistent with the granulomatous nature of Crohn’s disease. Surprisingly, ulcerative colitis—a non-granulomatous inflammatory bowel disease—has also been reported in a case of bilateral mammary abscesses, finally diagnosed as aseptic cutaneous abscesses [80]. In this latter observation, epithelioid granulomas within the breast tissue illustrate the potential difficulty in distinguishing Crohn’s disease from ulcerative colitis.

#### 5.1.2. GPA

Granulomatosis with Polyangiitis (GPA, formerly Wegener’s disease) is characterized by necrotizing granulomatous inflammation, usually involving the upper and lower respiratory tract, and necrotizing vasculitis, mainly affecting the small- and medium-sized vessels [81] (Figure 3D). Anti-neutrophil cytoplasm antibodies (ANCA), mainly directed towards proteinase 3 (PR3), are detected in many patients, although their presence is not essential to the diagnosis of GPA. Limited expression of GPA (especially affecting the upper or lower respiratory tract or orbit) may occur in the absence of detectable lesions of vasculitis. In these limited forms, ANCA positivity strengthens the diagnosis of GPA. The potential severity of the disease is illustrated by this case, published in 1971: a 40-year-old woman with a fast-growing right breast mass, whose biopsy revealed granulomatous mastitis [82], died six months later from lung and renal failure. At the time, destruction of the right breast and nipple had been observed, as well as destruction of the lower larynx and trachea at the post-mortem examination. ANCA blood tests were not yet available to facilitate the diagnosis.

Mammary involvement in GPA patients is rare: patients (mostly women) present with a uni- or bi-lateral breast mass, which is either painful or not and, more rarely, skin inflammation/ulcerations or nipple discharge. One case of breast involvement in a man was reported (40 years old, bilateral breast masses and skin ulcerations, small-vessel vasculitis and granuloma) [83].

In 2009, Allende and coll. reported 27 cases of GPA mastitis in their extensive literature review [74]. Available histological findings were breast vasculitis in 14 patients and granulomatous mastitis in seven patients. In most of the cases, systemic GPA symptoms preceded the onset of breast symptoms [84,85,86,87]. Our literature review found 9 cases of GPA revealed by breast disease, initially restricted to the breast [82,83,88,89,90,91,92,93,94]. In these cases, systemic symptoms occurred several weeks (3–24 weeks) after diagnosis of breast disease, and sometimes later (about 10 years) [90,91]. In this subgroup of patients with initial breast symptoms, one patient died (Table 1). Ren and coll. published an extensive review clustering all kinds of breast vasculitis. Breast GPA concerned 23 patients [95]. Two (out of 12 patients with available data) did not show ANCA positivity. Astonishingly, out of 22 patients with available data, they reported that 5 GPA patients were not treated by glucocorticoids (GC) and 6 of them did not receive any immunosuppressants. Three of them died.

Indeed, the therapeutic management of GPA requires the administration of immunosuppressive drugs (such as cyclophosphamide or rituximab) in addition to corticosteroid treatment, even in cases of limited GPA. Otherwise, severe life-threatening damage (such as an intra-alveolar hemorrhage of the lung or kidney failure) may occur. Consequently, the discovery of granulomatous inflammation or breast vasculitis within breast lesions must be urgently taken into consideration in conducting a systemic diagnosis procedure in order to adapt the treatment strategy. In this context, a test for circulating ANCA must be performed, specifically when the histological pattern is incomplete (without vasculitis) [96].

#### 5.1.3. EGPA

Eosinophilic Granulomatosis with Polyangiitis (EGPA, formerly Churg-Strauss syndrome) is another form of ANCA-associated small vessel vasculitis, in which eosinophilic infiltrates can be found within tissues. The clinical presentation includes hypereosinophilic asthma and lung infiltrates, whereas systemic vasculitis may affect the kidneys, peripheral nerves, and skin. EGPA localized to the breast was reported in 5 cases from literature, presenting as a breast tumor, bilateral mastitis, or nipple discharge [97,98,99,100,101]. Histopathological examination revealed eosinophilic infiltrates of the breast [98,99], eosinophilic infiltrates within vessel walls [99], panniculitis, or skin and deep tissue inflammation [97]. Breast symptoms revealed vasculitis in one of these cases [100].

#### 5.1.4. Sarcoidosis

Sarcoidosis is a systemic disease, mainly affecting lungs, and is characterized by non-caseating epithelioid-cell granulomas (Figure 3B), with neither vasculitis nor tissue necrosis. Breast sarcoidosis must be distinguished from two peculiar conditions: sarcoidosis-like reactions surrounding a breast cancer and breast cancer in sarcoidosis patients.

Breast sarcoidosis appears to concern fewer than 1% of sarcoidosis cases [75,102]. According to Ojeda and coll., 35 cases of breast sarcoidosis with histological documentation, all in women, were reported in literature from 1921 to 1997 [75]. Our literature search has found 30 other cases to date, also presenting as lumps in the breast [5,72,103,104,105,106,107,108,109,110,111,112,113,114,115,116,117,118,119,120,121,122,123]. Painless breast masses were the main presentation, sometimes associated with skin with an “orange peel” aspect. In some cases, breast involvement was asymptomatic and sarcoidosis had been diagnosed in the context of breast cancer screening as a public health strategy [5,113,114,115]. Systemic symptoms usually preceded breast localization, whereas breast involvement was the first symptom of sarcoidosis in 11 of these patients.

Granulomatous reactions mimicking sarcoidosis can also be observed in progressive cancer. Sarcoidosis-like reactions surrounding breast cancer have been reported [117,124]. This makes the diagnosis of sarcoidosis (affecting breast and/or lymph nodes) more complex when patients have previously developed breast cancer [121,125,126]. Sarcoidosis occurring after solid cancer treatment mostly follows breast cancer [126]. In these cases, sarcoidosis presented with lymph nodes, suggesting a recurrence of cancer, and required a histological analysis to rule it out. Systemic treatment of sarcoidosis was necessary in half of these cases. The authors suggest that sarcoidosis following solid cancer should be considered as a protective factor against a relapse of cancer due to the low rate of cancer recurrence in this population [126,127]. In any case, the relationship between sarcoidosis and cancer as well as the impact of breast cancer treatment on the pathophysiology of sarcoidosis have yet to be completely elucidated.

Foreign-body reactions can also result in granulomatous lesions following silicone implant rupture or leakage and must be distinguished from “true” sarcoidosis, which occurs in the absence of breast implant rupture [128].

#### 5.1.5. Granulomatous Mastitis Associated with IgG4-Disease

The wide spectrum of IgG4-related diseases includes orbit, lachrymal, and salivary glands, thyroid, liver, biliary tract, blood vessels, kidneys, retroperitoneal compartment, and skin [129]. As described in §3.6, breast involvement may also occur in this condition, but remains uncommon. Twelve cases of granulomatous mastitis associated with IgG4 disease were reported in literature [49,76,130]. Since the clinical presentation (breast mass) is unspecific, a biochemical and immunohistochemical search for IgG4 disease (within serum and tissue, respectively) should be performed when a case of breast mass remains unexplained, especially for those exhibiting tissue lymphocytic infiltrates and/or granulomatous inflammation.

### 5.2. Granulomatous Mastitis Associated with Other Autoimmune Diseases

Surprisingly, granulomatous mastitis may also be observed in certain patients with well-characterized connectivitis, which is not considered as a granulomatous condition (including those with systemic lupus [131], SjS [132], or rheumatoid arthritis [133]). As discussed, this association of granulomatous inflammation and connectivitis may be observed in a subset of patients, especially in those with SjS [72,132,134,135].

### 5.3. IdGM: “Idiopathic” or “Immunological” Granulomatous Mastitis?

Most cases of granulomatous mastitis are not linked to a specific, well-defined autoimmune disease and are, thus, considered as “idiopathic” [136,137,138]. Among these patients, neither the histological breast pattern nor the typical extramammary clinicobiological findings are consistent with the diagnosis of a systemic autoimmune disease. The clinical presentation consists of a single painful breast mass or multiple inflammatory masses, inflammatory skin, lymphadenopathy, or nipple discharge. As with other kinds of mastitis, the clinical presentation can mimic breast infection. In many cases, recurrent episodes occur, leading to several potentially tissue-damaging surgical biopsies.

In the case series of 20 patients from Saudi Arabia reported by Baslaim and coll. [139], IdGM involved fewer than 2% of benign breast conditions. The prevalence is apparently higher among Turkish, Middle-Eastern, North African, and South-East Asian patients than in European patients [136,137,140,141,142,143,144]. Recently, in an extensive review by Martinez-Ramoz and coll., 70 studies reporting idiopathic granulomatous mastitis were identified, involving 3060 patients with a median age of 36 years old. Thirty percent of these patients were from Turkey, followed by other Mediterranean countries (Morocco, Egypt, Israel), Iran, India, and the USA. Patients presenting with IdGM were all women and most of them had breastfed their children; usually, IdGM occurs shortly after breastfeeding (around 40 months), but a recent history of lactation or pregnancy is inconstantly related to the diagnostic period [140,143].

As suggested by its name, the etiologies of “idiopathic” granulomatous mastitis are not clearly identified: both endogenous and exogenous factors are involved. Among the exogenous factors, tobacco use has been incriminated [145] and, likewise, infectious agents. Indeed, Helal and coll. report 17% (6/65) of IdGM in Egyptian patients showing Gram-positive bacilli in their breast biopsies [146]. A prospective study suggests that treatment with rifampicine for 6–9 months led to a complete remission of IdGM in 30 patients after a 15-month follow-up [147]. It should be noted that most of the published case series of IdGM concern patients from countries where tuberculosis has a high prevalence rate [148]. *Corynebacterium kroppenstedtii* has also been involved in the pathophysiology of IdGM and in its recurrence [145]. These points are consistent with the role of potential infectious agents that are involved in the pathophysiology of IdGM.

Multiple endogenous factors may also contribute to the pathophysiology of IdGM. Local triggering factors, such as ductal ectasia or a breast abscess, may initiate the granulomatous process [141,149,150,151], leading to an accumulation of protein-rich secretions, ductal perforation, galactophoritis, and granuloma formation. Lactation, pregnancy, and oral contraceptives are also suspected as worsening factors [136,152,153]. In some patients, the efficacy of bromocriptine on the disease’s progression has been observed, tentatively confirming the role of hormonal status and, more precisely, prolactine on the onset of IdGM [138]. Granulomatous mastitis arising under risperidone treatment (through prolactine secretion) also confirms the role of hormonal status in the pathophysiology of IdGM [154].

Nevertheless, the “idiopathic” nature of IdGM is unclear in some reported cases. For example, extramammary symptoms frequently include arthralgia and erythema nodosum [151,155,156]. In addition, some patients have detectable circulating autoantibodies, such as Rheumatoid Factor, ANA, or anti-DNA antibodies [144,157], suggesting a role of autoimmunity in the pathophysiology of IdGM [144]. A particular profile of Human Leukocytes Antigen has recently been suggested in these patients [158]. Finally, Martinez-Ramoz and coll. reported that “autoimmune diseases” or “rheumatic conditions” were reported in 34% of patients included in their extensive review [148].

These elements, which suggest the key role of autoimmunity in the pathophysiology of IdGM, are sustained by the therapeutic strategies used. Indeed, although wide surgical excision still remains a therapeutic option [142,148,159], patients with recurrent IdGM have a good clinical response after steroid and immunosuppressive treatment [148,160]. A large retrospective series of 206 Iranian women of childbearing age (22–40 years old) highlights the positive effect of immunosuppressive treatment [138]. Indeed, while antibiotics failed in this population, 144/200 women improved with steroids (10–20 mg of prednisolone three times a day). A combination of methotrexate (7.5 to 10 mg a week) and steroids was given to patients with steroid-resistant diseases. This strategy was effective in 44/56 women. Postolova and coll. reported 18/19 patients with significant clinical improvement after 15 months of methotrexate treatment [161]. Topical steroids also appear to be effective in these patients [162]. Other smaller studies support the efficacy of immunosuppressive treatment on the course of this disease [141,151,156,163]. However, considering their potential side-effects, these surgical or immunosuppressive treatments should be proposed only after a period of observation: indeed, David and coll. reported recently that 112/120 IdGM resolved spontaneously after a mean period of 5.1 months [164].

Considering the reported cases and efficacy of immunosuppressive treatments, the immune system should be considered as a keyplayer, contributing to the pathophysiology of IdGM, as well as endocrine factors.

## 6. Vasculitis

Vasculitis is characterized by an inflammation of the blood vessels and can affect small, medium, or large vessels within the mammary glands. In such cases, and just like the other causes of mastitis, cancer must be sought because vasculitis can be observed surrounding cancer cells. Once cancer has been dismissed, other histological breast patterns (such as granuloma) should be checked. A complete examination and biological tests must be performed quickly in order to characterize the subtype of vasculitis and any potentially life-threatening damage (kidney, heart, or lung, for example). Superficial thrombophlebitis localized to the breast—Mondor’s disease’s—is not depicted here.

The type of targeted vessel is very helpful for conducting the diagnostic investigation: (i) small-vessel vasculitis leads to suspicion of granulomatous with polyangiitis, already depicted in §5.1.2, but may also indicate EGPA (§5.1.3) or micropolyangiitis (§6.3); (ii) medium-vessel vasculitis is suggestive of polyarteritis nodosa (§6.1); and (iii) large-vessel vasculitis will suggest giant cell arteritis (GCA) (§6.2). Both arterial and venous vasculitis can lead to the diagnosis of Behçet’s disease.

As described earlier, Allende and coll. performed a review of GPA-associated mastitis, but Ren and coll. recently published an extensive review of systemic vasculitis involving the breast [74,95]. Out of 67 cases found in the literature, Ren and coll. reported 34.3% of GPA mastitis, 4.5% of EGPA mastitis, 3% of MPA, 25.4% of polyarteritis nodosa (PAN), 25.4% of GCA, and 4.5% of Behçet’s disease. Three cases concerned undetermined vasculitis. Only two male cases were reported and the mean age of the population was 54.2 yo (+/− 14.7). Interestingly, extramammary signs were absent in 22.4% of the cases. Concerning the 28 cases of ANCA-associated vasculitis (AAV) with potentially life-threatening damage, the ANCA-test was available in 16 cases. Results were negative in 2 cases of GPA and 1 case of EGPA. This is of particular interest because besides the histological analysis, ANCA-testing is the cornerstone of AAV diagnostic criteria. Astonishingly, among the patients with available data, only 44/63 were treated with glucocorticoids (GV): 19 patients did not receive GC (5 with PAN, 5 with GCA and 6 with MPA, and 2 with Behçet’s disease). This is probably linked to a long delay in diagnosing vasculitis due to the atypical presentation of the disease. This under-treatment strategy must be considered along with the high mortality rate: six patients died (9.7%), five of whom were in the AAV subgroup.

### 6.1. Polyarteritis Nodosa

Polyarteritis nodosa (PAN) is characterized by vasculitis targeting medium-sized and small-sized vessels. The diagnosis is made when other forms of vasculitis (especially AAV) have been dismissed. Ren and coll. reported 17 cases of breast PAN and we found two other cases in our own literature review [83,165]. Eight of these 19 patients had no extramammary symptoms and 1 of them died. Four of them had ANCA positivity, although the final diagnosis was not AAV. One other case was reported after breast implants [166].

### 6.2. Giant Cell Arteritis

Ren and coll. reported 17 cases of GCA involving the breast. These were all women and 7 of them had bilateral symptoms [95]. None of them died. Six other cases were found in our review [167,168,169,170]. Six of these 23 cases showed no extramammary symptoms. The pathophysiological hypothesis concerning the breast’s involvement in GCA is based on anatomical findings: the internal mammary artery is a branch of the subclavian artery. Consequently, it may be affected by inflammation in other branches of the subclavian artery. Interestingly, among all these cases, we noticed three cases of concomitant breast cancer and GCA [167,168,171].

### 6.3. Microscopic Polyangiitis

One case of MPA with breast involvement was found [172]. This case concerned a 76-year-old woman with extramammary symptoms leading to a diagnosis of MPA. This presentation is different from other cases of MPA diagnosed after silicone breast implantation [173].

### 6.4. Behçet’s Disease

Behçet’s disease targeting the breast is very rare. Three cases have been reported, presenting as inflammatory breast tenderness or ulcers [174,175,176]. One other case of Behçet’s disease involving the breast was reported in a male patient with ductal ectasia rather than vasculitis (§4) [69].

## 7. Discussion

**Figure 4:**
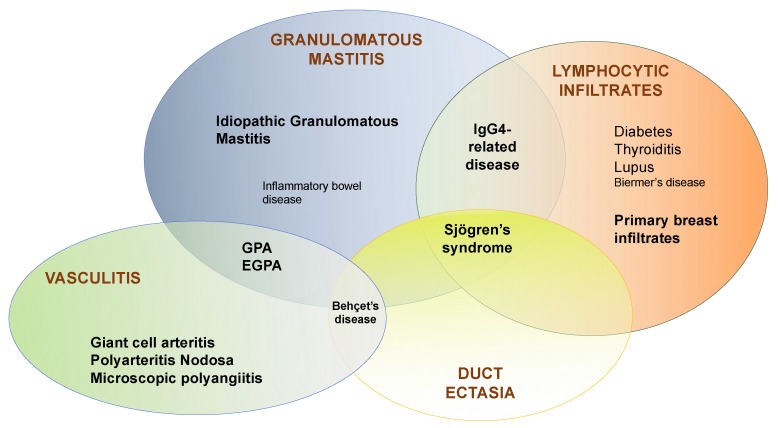
Breast biopsy histological patterns and corresponding defined auto-immune diseases. As described, IgG4-related breast disease, Sjögren’s syndrome-associated mastitis, breast GPA and EGPA, or Behçet’s disease can share several histological patterns. GPA, granulomatosis with polyangiitis; EGPA, Eosinophilic Granulomatosis with Polyangiitis; PAN, polyarteritis nodosa; GCA, Giant Cell Arteritis; IgG4, immunoglobulin type 4.

**Figure 5:**
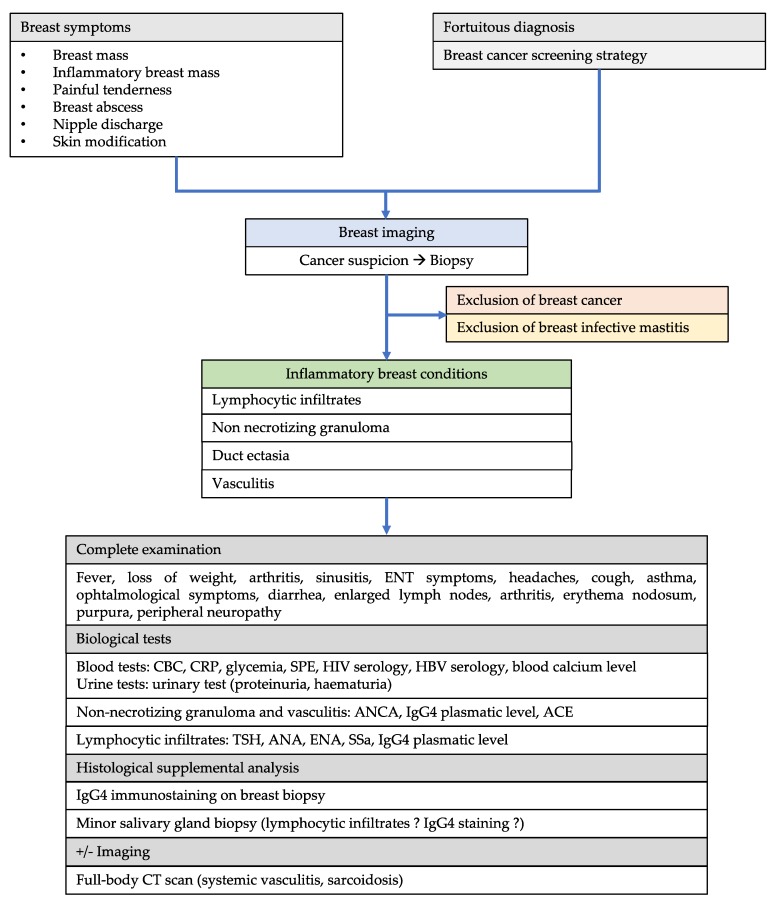
Proposed strategy for dealing with suspected inflammatory diseases of the breast. CBC, complete blood count; CRP, C-reactive protein; SPE, serum protein electrophoresis; HIV, human deficiency virus; HBV, hepatitis B virus; ANCA, Anti-neutrophil cytoplasm antibodies; ANA, antinuclear antibodies; ENA, extractable nuclear antigens; SSa, anti-Sjögren’s syndrome related antigen A antibody; ACE, angiotensin-converting enzyme.

To resume, mammary glands can be affected by a wide range of autoimmune diseases: (i) granulomatous diseases, such as sarcoidosis, GPA, or Crohn’s disease; (ii) connective tissue disease such as Lupus or SjS; (iii) organ-specific autoimmune diseases such as diabetes or thyroiditis; (iv) systemic diseases such as IgG4-related disease or systemic vasculitides. Physicians should follow a precise diagnostic pathway in order to make their diagnosis (Figure 4 and Figure 5). Indeed, histological features are insufficient for making the diagnosis since the same histological breast pattern may be shared by several autoimmune diseases (Table 1). Therefore, although they are rare, when the first symptoms target the breast, autoimmune diseases should be diagnosed as early as possible to avoid any potential life-threatening manifestations. The reasons why mammary glands are targeted are probably sustained by their peculiar characteristics, involving anatomical, histological, and functional features.

### 7.1. Intrinsic Characteristics of the Mammary Gland

The functional role of the mammary gland is not restricted merely to feeding newborns, but rather is directed towards immunomodulation. Besides having a crucial antibacterial role (especially through lysozyme and lactoferrin secretion), breast milk exhibits several immunomodulatory properties, assumed by IgA secretion, associated with lactobacillus, leukocytes, and macrophage secretion. Breastfeeding contributes to regulating the gut microbiota of newborns and maturing and regulating their immune system [177,178]. The histological features of the mammary gland support its physiological role of immunomodulation. Indeed, normal breast glands contain myeloid and lymphoid cells, located within the lobules rather than the stroma. Certain immunocompetent cells, such as T CD8+ lymphocytes, B lymphocytes, and dendritic cells, are integrated within the epithelium [6]. As an illustration of the importance of lymphoid components within the breast tissues, breast lymphomas are not rare, with a special pattern of mucosa-associated lymphoid tissue lymphomas [179]. Considering the major role of breast milk in regulating a newborn’s immune system and the importance of immune tissue within the breast gland, we thus conclude that the role of the breast’s mucosal immune system is crucial in the pathophysiology of autoimmune mastitis [180].

### 7.2. Environmental Factors

The activation of breast tissue cells (lymphocytes, macrophages, or dendritic cells) mostly results from the migration of cells from inducing sites of the mucosa immune system, especially gut-associated lymphoid tissue. Thus, environmental factors (gut microbiota, for example) that usually target gut lymphoid tissue are involved in activating the mucosa immune system, including mammary gland immune mucosa lymphoid tissue. Moreover, infectious diseases may trigger breast lymphoid tissue activation, especially during the breastfeeding period. Other infectious agents, such as viruses, could also infect a non-lactating mammary gland, similarly to what can happen within salivary or other exocrine glands. These infectious environmental factors are presumed to contribute to the onset of autoimmune mastitis.

### 7.3. Hormonal Factors

Hormonal status, especially plasma estrogen level, is a very classical contributor to the pathophysiology of autoimmunity. The variation of plasma estrogen levels has an impact on both the activity and functions of underlying breast-associated immune cells via estrogen receptor (ER) activation. For example, activating dendritic cells’ (DC) ER would lead to an imbalance of secretion towards a pro-inflammatory process (favoring interferon and IL-6 secretion and CD40, CD86, and MHCII molecule expression) [181,182]. Activating the dendritic cells’ ERs also appears to have a role in DC differentiation [182]. In a mouse model for lupus, ER was demonstrated to modulate plasmacytoid dendritic cell function and interferon activity [183]. In another model (C57BL/10.Q mice) [184], plasma estrogen levels may also have contributed to the recruitment of innate immune cells towards inflammatory sites. Plasma estrogen levels may also impact the function of targeted tissues themselves: for example, the salivary gland cells of SjS patients express functional ER exhibiting immunomodulatory properties (like INF-γ inducible ICAM-I expression) [185]. This hypothesis about the role of hormones in autoimmune mastitis is in line with epidemiological data, suggesting that women of childbearing age are more prone to developing autoimmune mastitis.

As described earlier, another potential hormonal key player in autoimmune mastitis is prolactin [152]. Although not fully demonstrated, prolactin has already been discussed as a potential player in certain autoimmune diseases such as lupus [186,187,188]. Breastfeeding, through prolactin secretion (including synovial prolactin secretion), seems to be a period of higher risk for rheumatoid arthritis or lupus flares [189,190]. In lupus patients, the immunosuppressive effect of T regulator cells over T effector cells is apparently modulated by prolactin secretion [191]. In addition, in psoriatic arthritis and rheumatoid arthritis patients, prolactin production seems to be increased within the macrophages of synovial fluid, while prolactin receptors seem to be upregulated on synovial tissue [189]. Taken together, the potential immune effects of estrogen and/or prolactin hormones on breast cells or the breast’s underlying immune system may lead to an imbalance towards a pro-inflammatory axis, stimulating the onset of autoimmune mastitis.

### 7.4. The Role of Mammary Epithelial Cells

Mammary epithelial cells are probably key players in the interaction between environmental factors and immune system. Firstly, the epithelial cells may act as antigen-presenting cells, exposing infectious antigens on their surface, notably after TLR activation by infectious agents. This point has been described in Sjögren’s syndrome in which salivary gland epithelial cells (SGECs) express class II HLA molecules, costimulatory molecules, and toll-like receptors (TLRs) and consequently act as antigen-presenting cells, even after long-term ex vivo culture [192,193]. Secondly, epithelial cells are in close connection with the underlying mucosa-associated lymphoid tissue in the breast (i.e., intra-epithelial dendritic cells and T CD8+ lymphocytes) and exhibit immunomodulatory properties. For example, SGECs from SjS patients may promote in vitro T CD4+ differentiation into T follicular helper cells [194]. Under interferon stimulation (triggered by innate immunity and viruses), SGECs secrete high amounts of the B-cell activating factor (BAFF) involved in B-cell activation [194,195]. We may note that in breast cancer, breast tumor cells develop immunomodulatory properties in order to modulate anti-tumoral immune system strategy, such as PD1-L expression [196]. Thirdly, epithelial breast cells share certain self-antigens involved in autoimmune diseases with other epithelial cells from other targeted tissues. For example, the SSa antigen in SjS is exposed both in salivary and mammary epithelial cells [35,197]. At least, mammary duct self-antigens may exhibit a cross-reactivity towards other autoantibodies. This mechanism has already been described in a long-lasting Type 2 diabetic patient treated with insulin, in whom anti-insulin autoantibodies cross-reacted with breast ductal epithelium, leading to ductitis and mastitis [30]. This mechanism of cross reactivity could also be involved in the cases of concomitant breast cancer and vasculitis [167,168]. Notably, Kafantari and coll. reported the case of a 74 year-old-woman with concomitant breast ductal invasive carcinoma and GCA [171]. In this case, GCA symptoms disappeared after tumor removal.

## 8. Conclusions

When considering inflammatory symptoms of the breast or inflammatory histological patterns discovered after a biopsy performed in a breast cancer diagnosis strategy, physicians must be aware of potential autoimmune mastitis. This clinical condition has a diagnosis of exclusion, requiring breast infection and/or cancer to be excluded. Furthermore, an early diagnosis of autoimmune mastitis can avoid unnecessary repeated breast biopsies or surgery, which are potentially devastating. All types of autoimmune diseases can target the mammary glands: organ-specific diseases (diabetes, thyroiditis) or systemic diseases such as connective tissue disease (systemic erythematosus lupus, Sjögren’s syndrome), vasculitides (granulomatosis with polyangiitis, eosinophilic granulomatosis with polyangiitis), granulomatous diseases (sarcoidosis, Crohn’s disease), or IgG4-related diseases. Moreover, the breast may be the first target of a potential life-threatening systemic disease such as ANCA-associated vasculitis. An early diagnosis is therefore required in order to initiate immunosuppressive treatments, thereby improving the general prognosis of the disease.

## Figures and Tables

**Table 1:** Clinical, immunological, histological, and reported outcomes of the main autoimmune diseases targeting the breast. GPA, granulomatosis with polyangiitis; EGPA, Eosinophilic Granulomatosis with Polyangiitis; Eosino., Eosinophilic; PAN, polyarteritis nodosa; GCA, Giant Cell Arteritis; SjS, Sjögren’s syndrome; IGM, Idiopathic Granulomatous Mastitis; IgG4, immunoglobulin type 4; ANCA, Anti-neutrophil cytoplasm antibodies; ANA, antinuclear antibodies; ENA, etractable nuclear antigens; SSa, anti-Sjögren’s syndrome related antigen A antibody; ACE, angiotensin-converting enzyme; TAP, Temporal artery biopsy; IH, immunohistochemical staining.

	GPA	EGPA	Behçet’s Disease	PAN	GCA	Sarcoidosis	IgG4-RD	Lupus	SjS	IGM
**Clinical presentation**										
Fortuitous (breast cancer screening strategy)										
Breast mass (uni- or bi-lateral)										
Inflammatory breast mass; painful tenderness; breast abscess										
Nipple discharge										
Skin ulcers, skin modification										
**Immunological features (blood tests)**	ANCA	ANCA				ACE	IgG4	ANA	ANA, ENA, SSa	ANA
**Breast histological features**										
Lymphocytic infiltrates										
Hyaline fat necrosis										
Panniculitis										
Vasculitis										
Non necrotizing granuloma										
Duct ectasia										
Fibrosis										
Specific features		Eosino. infiltrates			TAB		IH: IgG4 staining	IH: IgG, C3 deposit		Negative microbial culture
**Breast initial or unique symptoms**										
**Potential life-threatening damages**										
**Reported related death**										
